# Three-dimensional nonlinear photonic crystal in naturally grown potassium–tantalate–niobate perovskite ferroelectrics

**DOI:** 10.1038/s41377-020-00427-z

**Published:** 2020-11-24

**Authors:** Chang Li, Xuping Wang, Yang Wu, Fei Liang, Feifei Wang, Xiangyong Zhao, Haohai Yu, Huaijin Zhang

**Affiliations:** 1 State Key Laboratory of Crystal Materials and Institute of Crystal Materials, Shandong University, Jinan, 250100 China; 2 Advanced Materials Institute, Qilu University of Technology (Shandong Academy of Sciences), Jinan, 250014 China; 3 Key Laboratory of Optoelectronic Material and Device, Department of Physics, Shanghai Normal University, Shanghai, 200234 China

**Keywords:** Photonic crystals, Photonic devices

## Abstract

Since quasi-phase-matching of nonlinear optics was proposed in 1962, nonlinear photonic crystals were rapidly developed by ferroelectric domain inversion induced by electric or light poling. The three-dimensional (3D) periodical rotation of ferroelectric domains may add feasible modulation to the nonlinear coefficients and break the rigid requirements for the incident light and polarization direction in traditional quasi-phase-matching media. However, 3D rotating ferroelectric domains are difficult to fabricate by the direct external poling technique. Here, we show a natural potassium–tantalate–niobate (KTN) perovskite nonlinear photonic crystal with spontaneous Rubik’s cube-like domain structures near the Curie temperature of 40 °C. The KTN crystal contains 3D ferroelectric polarization distributions corresponding to the reconfigured second-order susceptibilities, which can provide rich reciprocal vectors to compensate for the phase mismatch along an arbitrary direction and polarization of incident light. Bragg diffraction and broadband second-harmonic generation are also presented. This natural nonlinear photonic crystal directly meets the 3D quasi-phase-matching condition without external poling and establishes a promising platform for all-optical nonlinear beam shaping and enables new optoelectronic applications for perovskite ferroelectrics.

## Nonlinear photonic crystals: controlling light from any which way

A three-dimensional nonlinear photonic crystal that manipulates light incident from any direction could allow new types of beam shaping for optoelectronic devices. In certain crystals, atoms arrange in mesoscopic structures called supercells, which cause light to interfere in useful ways. Generally, such crystals can only affect light beams that are incident from particular directions, and with certain polarizations. Now, Haohai Yu at Shandong University in Jinan, China, and co-workers have grown crystals of potassium tantalate niobate, with the constituent atoms in a critical ratio that provides a Rubik’s-cube-like structure with supercells 3–10 µm in size. The researchers observed rotating ferroelectric polarization and second harmonic generation in laser beams incident from all three dimensions (*x*, *y*, and *z* directions). They hope that the crystal could provide a useful platform for applications such as terahertz radiation and holography.

## Introduction

The macroscopic physical properties of condensed matter are associated with constituent functional groups, concentration gradients, as well as their hierarchical spatial arrangement^1–5^. An example of a counterintuitive mesoscopic phase that naturally mimics standard solid-state structures but on scales that are thousands of times larger is a supercell^6^. In perovskite ferroelectrics, microscopic metal-centered octahedrons are the main contributor to nonvolatile polarization, and mesoscopic ferroelectric domains are the most attractive regulation factor for the design of ferroelectric supercells, including the domain size, poling direction, and flexible nanodomains^7–10^. As a paradigm, periodic and quasi-periodic ferroelectric domains with inverse polarization have been designed to make meta-materials^11^, especially for photonic and phononic crystals^12–14^. By employing the interference of light or acoustic waves in designed submicrometer and micrometer regions, various intriguing properties for the incident photons or lattice vibrations emerge in these artificial super-crystals^15,16^, such as harmonic generation, dielectric abnormality, and polariton excitation. In nonlinear photonic ferroelectrics, the laser spectral range is widely extended by quasi-phase-matching due to periodic domain inversion that corresponds to a change in the signs of the nonlinear susceptibility and supplies the reciprocal vectors *G* to compensate for the phase-mismatch Δ*k*. To date, the distribution of reciprocal vectors in one, two, and three dimensions (1D, 2D, and 3D, respectively) have been demonstrated in several ferroelectric nonlinear crystals^4,17–19^.

The ferroelectric polarization direction determines the quasi-phase-matching condition. Regardless of the previous 1D, 2D, or 3D quasi-phase-matching examples, the Δ*k* = 0 condition has strict limitations for the crystal cutting direction and incident light polarization for efficient nonlinear energy conversion. For example, in periodic poled LiNbO_3_ crystals with up–down ferroelectric domains, the incident light direction should be along the x-cut crystal to take advantage of its largest nonlinear susceptibility *d*_33_, and the polarization of fundamental and second harmonic light is also limited in the *z* direction^18^. Therefore, breaking such constraints has a profound impact on nonlinear photonics. The present predicament could be addressed by the spatial rotation of ferroelectric domains since these composite rotated domains could be useful for different types of phase matching with additional 3D reciprocal vectors along with arbitrary directions. However, due to the limitations of traditional poling techniques, a nonlinear optical medium with 3D rotated ferroelectric domains is not feasible and has not been fabricated to our knowledge.

**Fig. 1:**
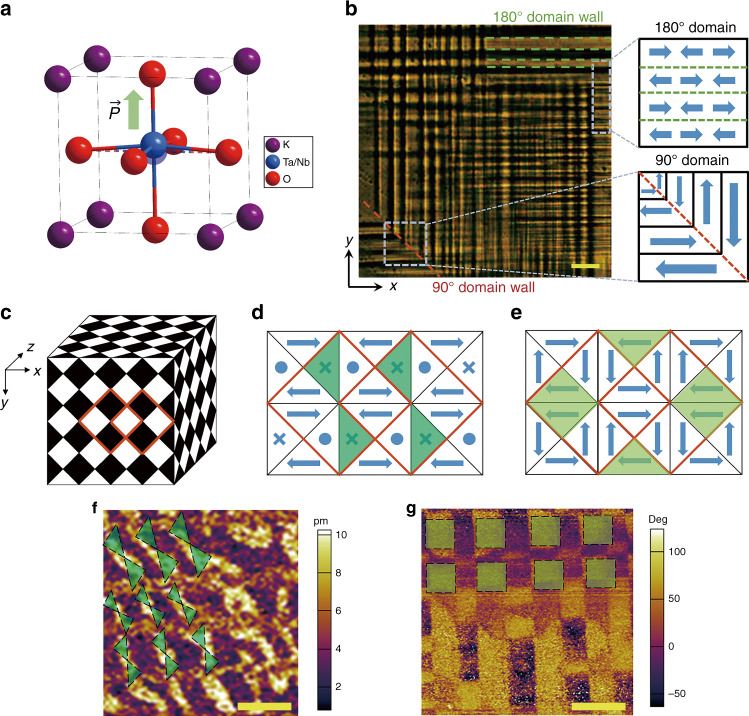
Crystal structure and ferroelectric domain distribution of KTN. **a** Crystal structure of tetragonal KTN. **b** Polarizing microscope image of the KTN crystal along the *x*–*y* plane; the scale bar is 25μm. The red dotted line represents the 90° domain wall, and the green dotted line represents the 180° domain wall. The blue arrows represent the spontaneous polarization direction. **c** Diagram of natural Rubik’s cube-like domain structures in the KTN crystal. **d** Structural model of the ferroelectric domain (out-of-plane) in the KTN *x*–*y* plane. The solid forks and points represent inside and outside polarization. **e** Structural model of the ferroelectric domain (in-plane) in the KTN *x*–*y* plane. **f** Vertical PFM amplitude and **g** lateral PFM phase images of the KTN *x*–*y* plane in the same region. The scale bar is 2μm

The Curie temperature is a crucial factor for regulating the internal polarization of ferroelectric domains, and discrete domains are reconfigured near the Curie temperature^6^. Perovskite potassium tantalite niobate, KTa_*x*_Nb_1−*x*_O_3_ (KTN), crystals feature a continuous solid solution with an adjustable Ta/Nb ratio (Fig. 1a). Impressively, their Curie temperature varies with a fluctuating Ta/Nb ratio^20^. The phase transition from the ferroelectric orthorhombic to tetragonal phase occurs at approximately 300 K for *x* ≈0.5 with the Curie temperature; the phase transition from the ferroelectric tetragonal to paraelectric cubic phase occurs at 400 K. Therefore, composite ferroelectric domains are formed at room temperature with a suitable Ta/Nb ratio, and the polarization directions are distributed in the three dimensions inherited from the cubic symmetry. Then, new physical properties emerge, including a large quadratic electro-optic coefficient^21^, large broadband refraction^22^, and diffractionless light waves^23^. Inspired by this point, we show an exotic addition to a KTN crystal by selecting a suitable Ta/Nb ratio and produce the first natural three-dimensional nonlinear photonic crystal with spatially rotated ferroelectric domains and modulated quadratic susceptibility. Both linear and nonlinear optics are studied, and broadband second-harmonic generation from 450 to 600 nm is presented on the KTN crystal.

## Results

### Crystal growth and basic characterization

The KTN crystal herein with a tetragonal phase (*P*4mm space group) at room temperature was grown by the Czochralski method^20^ (see Supplementary Materials). X-ray photoelectron spectroscopy (XPS) measurements show a semiquantitative Ta/Nb ratio of 0.56:0.44 (Fig. S1), suggesting a chemical formula of KTa_0.56_Nb_0.44_O_3_. The hysteresis P–E loop (Fig. S2) displays standard ferroelectric behavior, where the positive and negative saturated polarizations are 16.98 and −16.69 μC/cm^2^, respectively, and the positive and negative coercive fields are 3.68 and −2.44 kV/cm, respectively. Differential scanning calorimetry (DSC) measurements (Fig. S3) depict a near room-temperature Curie temperature of approximately 40 °C, which is much lower than that of LiNbO_3_ (~1140 °C) and Ba_0.77_Ca_0.23_TiO_3_ (~107 °C). This relatively low Curie temperature in KTN could be attributed to the small B site off-center distance in the oxygen octahedrons. Accordingly, the discrete ferroelectric domains in the KTN crystal would compete energetically, thereby reconstructing to form a 3D ferroelectric supercell with spatially distributed polarization.

Ferroelectric domains in the as-grown KTN crystal were observed by polarizing microscopy. As recorded in Fig. 1b, 90° domain walls (red dotted line) and 180° domain walls (green dotted line) can be clearly observed, which indicates the in-plane polarization direction in the horizontal and vertical domains. The statistical domain structure with a length of approximately 5 μm accounts for the most. This composite ferroelectric domain structure in the super cell is composed of 90° and 180° domains with different polarization directions. The spontaneous polarization of the KTN crystal, a typical perovskite ferroelectric crystal, is along the fourfold axis of the primitive cell with six possible directions, which makes the ferroelectric comprising KTN have 180° domains and 90° domains in different directions at the same time. Near the Curie point, 180° domains and 90° domains are recombined into a new periodic structure in the 3D space of the KTN crystal, where the domains are inlaid with each other to form a supercell. The supercells are periodically arranged in crystals with Rubik’s cube pattern (Fig. 1c), so the ferroelectric domains in the supercell can form a new type of three-dimensional structure. As shown in Fig. 1d, e, there is up–down ferroelectric polarization along the *x*, *y*, and *z* directions simultaneously. In other words, this polarization distribution allows quasi-phase-matching of the polarized incident light along with three directions. This natural 3D ferroelectric supercell is different from the previously reported artificial 3D nonlinear photonic crystals comprising LiNbO_3_ and Ba_0.77_Ca_0.23_TiO_3_. To our knowledge, this is the first spontaneous 3D nonlinear photonic crystal without any electric and light poling fabrication.

The piezo-response force microscopy results also support our statement. Figure 1f, g reveals the investigations of the domain structure of KTN (also see Fig. S4), and the data show the polarization distributions for both the vertical (out-of-plane) and lateral (in-plane) components. The bright and dark contrasts in the amplitude image reflect the relative magnitude (phase) of the piezoelectric response for each domain. Clearly, there are periodic domain structures in vertical and lateral images at the same time. The dark green regions in Fig. 1d represent the bright spot in Fig. 1f, and the green blocks in Fig. 1e are related to the bright spot in Fig. 1g. The out-of-plane polarization exhibits an hourglass-shaped configuration, while the in-plane polarization enables quasi-square alignment in the KTN *x*–*y* plane. Therefore, this demonstrates the existence of periodic out-of-plane and in-plane polarization spontaneously. This exotic and stable ferroelectric domain arrangement provides an attractive optical response in the KTN crystal.

### Bragg diffraction and second-harmonic images

**Fig. 2:**
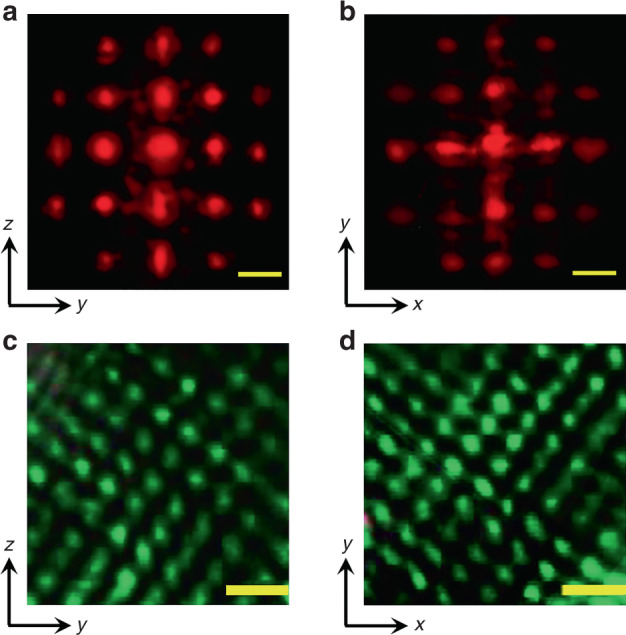
Linear and nonlinear optical response of the KTN crystal. **a**, **b** The Bragg diffraction spots from the x-cut and z-cut KTN samples (the scale bar is 2cm). **c**, **d** The second harmonic (SH) microscopic pattern of the x-cut KTN sample and z-cut KTN sample (the scale bar is 25μm)

Next, the linear and nonlinear optical responses of the KTN photonic crystals with different cutting directions were evaluated. According to Fresnel–Kirchhoff diffraction theory, the distribution of the Fraunhofer diffraction field observed in the far-field can be regarded as the intensity distribution of the inverted lattice vector space of the diffraction grating. Figure 2a, b shows the diffraction spots from the laser light passing through the x-cut and z-cut KTN samples, respectively. The 632.8 nm laser generates strong anisotropic diffraction through the crystal in the far-field. They appear as an optical analog of X-ray diffraction in low-temperature solids. The diffraction pattern clearly shows first- and second-order Bragg diffraction, suggesting that the internal structure of the KTN crystal has multiple dimensions in both the x-cut and z-cut samples simultaneously. As stated in Fig. 1, the KTN crystal has a multi-dimensional periodic structure, including in the 45°, 90°, 135°, and 180° directions and in the *x*–*y* and *y*–*z* planes. According to ferroelectric crystallography, the walls of 90° domains in ferroelectric crystals are 45° from the direction of the crystallographic main axis, while the domain walls of 180° domains are either parallel or perpendicular to the crystallographic main axis. The KTN crystal can have periodic ferroelectric domain structures in six directions simultaneously in the crystallographic plane only when the 90° and 180° domains exist simultaneously in the ferroelectric supercell. These ferroelectric volume microstructures cause an index of refraction periodic oscillation, which can diffract light linearly. Therefore, the observed Bragg diffraction is consistent with multiple periodic structures in the KTN crystal. The cell constant for this kind of super-crystal is scaled to the wavelength of the visible laser and corresponds to 10^4^ times the primitive cell constant. According to Bragg diffraction theory, the angle formed by the scattered light from the first Bragg diffracted spot to the incident wave vector fulfils the diffraction condition 2*d* sin *θ* = *λ*. Considering that the first-order diffraction spots are extended into a long strip, the available supercell constant *d* is calculated to be from 3.3 to 7.3 μm. This is consistent with a previous report for Li:KTN^6^ with a periodic dielectric volume microstructure of 5.5 μm that was able to diffract light linearly. Therefore, the fabrication of three-dimensional supercells in the KTN crystal was proven by the diffraction spots from the reciprocal vector space.

In ferroelectric crystals, the positive and negative nonlinear coefficients of crystals change with the polarization direction of the ferroelectric domain. The 180° periodic ferroelectric domain pattern provides an effective reciprocal vector to compensate for the phase mismatch caused by refractive index dispersion, thus enabling the effective growth of the second harmonic intensity. However, 90° ferroelectric domains cannot provide nonlinear coefficient modulation to compensate for a phase mismatch. Therefore, the domain structure of a supercell can be clearly observed through an SHG microscope in the near field. By applying a femtosecond laser (1030 nm, 2 MHz repetition rate, and 250 fs pulse width) incident to the KTN crystal, the SH light is captured by the microscope. As shown in Fig. 2c, d, the inverted periodic 180° ferroelectric domains can provide RLVs along two orthogonal directions, which leads to effective SH light (green spot). However, the boundary of the orthogonal ferroelectric domain region along the 45° direction is the 90° domain walls, which provide no significant reciprocal lattice to produce a high SH intensity, thereby resulting in a dark region (Fig. S5). Therefore, the green spot in the SH image represents the 180° domain region, while the dark region represents the 90° domain region. Similar to that for Bragg diffraction, the SH image also has a multi-dimensional periodic structure in the *x*–*y* and *y*–*z* planes (45°, 90°, 135°, and 180° directions), which is consistent with the ferroelectric domain analysis in Fig. 1. Statistical calculations (Fig. S6) show that the narrowest and widest supercells have widths of 3.4 and 9.6 μm, basically covering the result calculated by Bragg diffraction. Therefore, the mesoscopic structure of the KTN crystal in real lattice space was again demonstrated by SH microscopy.

### 3D quasi-phase-matching in KTN crystals

The KTN crystal structure belongs to the tetragonal 4 mm point group, and the nonlinear optical coefficient tensor under Kleiman symmetry can be shown as

1 $$d_{ij} = \left( {\begin{array}{*{20}{c}} 0 & 0 & 0 \\ 0 & 0 & 0 \\ {d_{31}} & {d_{31}} & {d_{33}} \end{array}\begin{array}{*{20}{c}} 0 & {d_{15}} & 0 \\ {d_{15}} & 0 & 0 \\ 0 & 0 & 0 \end{array}} \right)$$

where the nonlinear optical coefficient^24^ is *d*_15_ = 25 pm/V, *d*_31_ = 20 pm/V, and *d*_33_ = 84 pm/V. Because of the modulation of the ferroelectric domain to the nonlinear coefficient and the three-dimensional distribution of the ferroelectric domain, the nonlinear susceptibility coefficient is also distributed periodically in three-dimensional space in the KTN crystal. Using the exponential representation for rotations, the rotation matrix *R*_*i*_ can be shown as $R_i = e^{\theta _i}$, and *θ*_*i*_ is expressed as

2 $$\theta _i = \left( {\begin{array}{*{20}{c}} 0 & { - \theta _z} & {\theta _y} \\ {\theta _z} & 0 & { - \theta _x} \\ { - \theta _y} & {\theta _x} & 0 \end{array}} \right)$$

where *θ*_*x*_, *θ*_*y*_, and *θ*_*z*_ are rotating angles along the *x*, *y*, and *z* principle axes, respectively. Then, the transformed nonlinear optical coefficient tensor $d{\prime}_{ij} = R_id_{ij}R_i^T$, where $R_i^T$ is the transpose matrix of *R*_*i*_. For the KTN crystal, the rotation angles are 90°, 180°, and 270°, and the transformed $d{\prime}_{ij}$ is depicted in the Supplementary Materials.

**Fig. 3:**
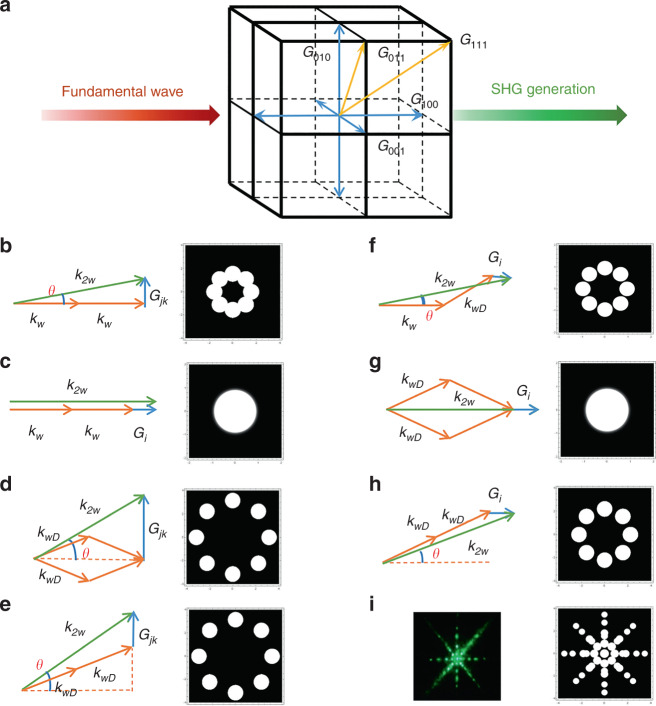
SHG spots from a 3D KTN crystal generated experimentally and by simulation. **a** Schematic of the 3D RLVs in the KTN crystal. **b** SHG spots produced by Raman–Nash diffraction of the 2D QPM. **c** SHG spots produced by collinear QPM. **d**, **e** SHG spots from the QPM produced by the interaction between the fundamental diffraction wave and 2D RLVs in the *y*–*z* plane. **f**–**h** SHG spots from the QPM produced by the interaction between the fundamental diffraction wave and RLV along the *x*-axis. **i** Comparison between the simulated and experimental results

In addition to spatially modulated nonlinear susceptibility, the three-dimensional ferroelectric polarization distribution also supplies a rich three-dimensional reciprocal lattice vector (RLV) to meet phase-matching conditions in second-harmonic generation. As shown in Fig. 3a. The available RLVs can be expressed as

3 $$\overrightarrow G _{lmn} = l\overrightarrow G _{100} + m\overrightarrow G _{010} + n\overrightarrow G _{001}$$

where *l*, *m*, and *n* are integral numbers. $\overrightarrow G _{100}$, $\overrightarrow G_{010}$, and $\overrightarrow G_{001}$ represent different RLVs, where the vector directions are along the *x*-axis, *y*-axis, and *z*-axis, respectively. Thus, the KTN crystal provides the capacity to produce 3D quasi-phase-matching without any artificial poling. The double-frequency phenomenon is completely different from ordinary nonlinear Raman–Nash diffraction and Bragg diffraction and resembles the superposition of multiple quasi-phase-matching.

In theory, we first consider the SHG spot distribution of the KTN crystal only under nonlinear conditions. Supposing that the fundamental laser beam with a wavelength of 1064 nm has a Gaussian distribution, the theoretical SHG pattern can be calculated. The computational details are depicted in Supplementary Materials. Figure 3b illustrates the SHG spot generated by the quasi-phase-matching effect of Raman–Nash diffraction when the fundamental wave vector is not collinear with the superlattice RLVs. The QPM condition is expressed as

4 $${\Delta}k = k_{2w} - 2k_w - G_{j,k} = 0$$

where *G*_*j,k*_ is the linear superposition of basic vectors $\overrightarrow G _{010}$ and $\overrightarrow G _{001}$ and compensates for the phase mismatch along with different directions. Figure 3c shows the quasi-phase-matching situation when the fundamental and doubling frequency light is collinear with the superlattice RVLs. In addition, the KTN supercell also provides periodic grating in the 45°, 90°, 135°, 180°, and directions as well as superlattice RLVs in the 3D space of the KTN crystal. Considering the three-dimensional periodic structure of the KTN crystal and the obvious diffraction effect, the fundamental frequency light produces a variety of diffracted light when it is incident on the crystal. The fundamental frequency diffracted light also achieves quasi-phase-matching associated with the super lattice RLVs in different directions and generates the second harmonic light. Figure 3d–h shows the SHG spot generated by the interaction between the diffracted fundamental frequency light and the superlattice RLVs in different directions. The simulated angles for Fig. 3b–h are listed in Table S1. To summarize the above situation, the total SH spot obtained by 3D quasi-phase-matching in the KTN crystal is a fourfold pattern, which is in good agreement with the experimental results (see Fig. 3i). This also supports the existence of 3D ferroelectric supercells in the KTN crystal.

**Fig. 4:**
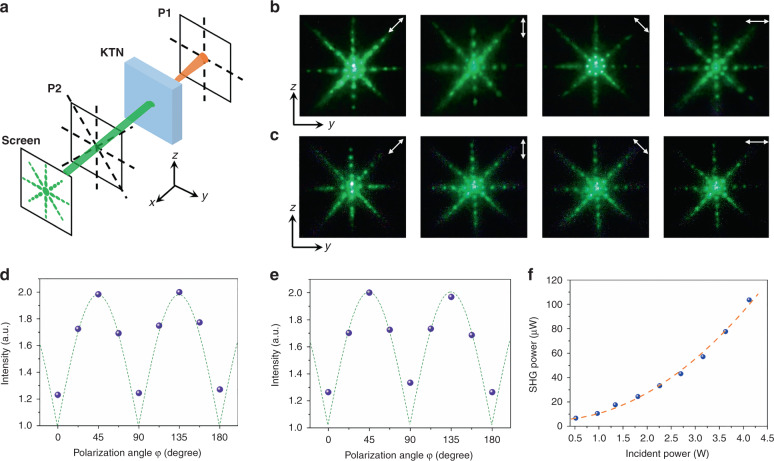
SH intensity distribution image for different polarizations. **a** Experimental apparatus for the 3D QPM SHG experiment. **b**, **c** SHG spots in different polarization states when the polarization direction of the incident fundamental light is along the *y*-axis (**b**) and *z*-axis (**c**). **d**, **e** Relative intensity of SHG in different polarization states when the polarization direction of incident fundamental light is along the *y*-axis (**d**) and *z*-axis (**e**). **f** The relationship between the fundamental power and SH power

Finally, the 3D quasi-phase-matched SHG pattern and intensity were measured for various polarizing states. The experimental apparatus is displayed in Fig. 4a. P1 and P2 are the polarizer and analyzer before and after the KTN sample, respectively. The SH spot images are shown in Fig. 4b, c, with the polarization of the incident laser along the *y*-axis and *z*-axis, respectively. In our KTN nonlinear photonic crystal, the limitation on incident light polarization in the QPM condition is broken. For the x-cut KTN crystal, there are periodic up-and-down ferroelectric polarizations along the *y* and *z* directions at the same time (see Fig. 1d, e). Therefore, the SHG spots with a multi-polarized SH wave are the same when the fundamental light is polarized along the *y* and *z* directions. This reveals that the 3D rotating ferroelectric supercells provide spatial nonlinear coefficient modulation, which breaks the strict limitations on incident light polarization and crystal direction in traditional QPM crystals that only have inverse polarization along one direction. From this viewpoint, the KTN crystal exhibits great improvements for practical applications.

As mentioned above, ferroelectric domains can be divided into four regions in a square supercell whose polarization directions are perpendicular to each other, namely, 90°, 180°, 270°, and 360°. We also give mathematical derivations of the spatial SHG coefficient distribution and polarization–intensity relation (see Supplementary Materials). The SHG beam has two polarization states concurrently, and the total SHG intensity is the superposition of two polarization intensities. Therefore, the SHG intensity PSHG should satisfy the relation of $P_{\rm{SHG}} \propto \left( {\left| {\cos \varphi } \right| + \left| {\sin \varphi } \right|} \right)^2$, where *φ* is the angle between polarizer *P*2 and the *z*-axis. As shown in Fig. 4d, e, the SHG intensity reaches the largest value when the angle *φ* equals 45° and 135° and gradually decreases to the minimum when the angle *φ* equals 0° and 90°. This is totally consistent with our analysis and calculations. The similarity of the nonlinear performance of different polarized incident lasers along different directions indicates that the ferroelectric domain rotation in KTN eliminates the strict direction limitation that controls previous 1D, 2D, and 3D phase matching^25^. Figure 4f depicts the relationship between the incident fundamental power at a wavelength of 1064 nm and the generated collinear SHG power. When the pump power is 4.12 W, the SHG intensity is 103.67 μW, resulting in a collinear SHG conversion efficiency of 2.52 × 10^−5^. This is comparable to that in a 3D nonlinear photonic BCT crystal^19^ but slightly smaller than that of a 3D nonlinear LiNbO_3_ crystal^18^. This may be attributed to extra scattering on complex domain walls in KTN. Such a conversion efficiency could be further improved by optimizing the experimental apparatus, crystal quality, and incident power. Furthermore, rational control of the period and duty cycle could give rise to rich nonlinear patterns in 3D KTN photonic crystals.

In addition to 1064 nm, our KTN crystal also supports broadband SHG generation with fundamental waves ranging from 900 to 1200 nm. As shown Fig. S7, wide-range SHG signals at 450 nm (blue), 530 nm (green), 570 nm (yellow), and 600 nm (red) are observed. We also measured the SHG output power with fundamental waves at 900, 1040, and 1160 nm. The quadratic relationship between the incident power and SHG power indicates that KTN could achieve broadband QPM conditions owing to its broad range of ferroelectric domains.

## Discussion

As an engineered material with modulated quadratic nonlinear susceptibility, this 3D nonlinear photonic KTN crystal can be used extensively in many scientific and industrial fields that require the generation and control of light at new frequencies. It breaks strict constraints on incident light polarization and crystal direction for QPM conditions over a wide spectral range. It enables new manipulation schemes for nonlinear optical interactions and enables additional applications, including a cascaded QPM for different nonlinear processes^26^, nonlinear Talbot imaging^27^, on-chip entangled light sources^28^, terahertz radiation^29^, 3D nonlinear holography and beam shaping. The last one has been partially demonstrated in 3D LiNbO_3_ and Ca_0.28_Ba_0.72_Nb_2_O_6_ nonlinear photonic crystals^12,13^. The rich coherent light sources may also be applied in fundamental atomic, molecular, and optical physics especially advanced scientific instruments with wide spectrometers and high resolution.

In summary, the first as-grown 3D nonlinear photonic KTN crystal was presented that was formed by the recombination of composite ferroelectrics and spatial rotation of domains. Both linear and nonlinear optical responses demonstrated periodic mesoscopic supercells. The exotic SHG spot and efficient SHG generation revealed rich RLVs in the natural KTN, which could break the strict requirements for the incident light and crystal direction in traditional poling nonlinear photonic crystals. It is readily compatible with the laser writing technique, which suggests promising opportunities to create hierarchical nonlinear optical modulation. Therefore, this 3D nonlinear photonic crystal in perovskite ferroelectrics should find a wide variety of applications in optical communications, nonlinear imaging, and on-chip signal processing.

## Materials and methods

### Materials and characterization

The KTN crystals utilized in this experiment were grown by the Czochralski method. The K_2_CO_3_, Ta_2_O_5_, and Nb_2_O_5_ chemical reagents with 99.99% purity were used as raw materials for the crystal growth. XPS was performed using a monochromatic aluminum target Kα source (Thermo Fisher Scientific Escalab-250). The hysteresis loop of the KTN crystal was measured using a ferroelectric test system (RT-Precision LC, Radiant Technology) at room temperature. The Curie temperature of the KTN crystal was measured by a Labsys^TM^ EVO DSC thermal analyzer.

### Second-harmonic generation experiment

A piece of an x-cut KTN crystal with dimensions of 0.4 mm × 4 mm × 4.5 mm (*x* × *y* × *z*) was employed, and the two *y*–*z* faces were polished. An Nd:Y_3_Al_5_O_12_ laser (1064 nm, 20 kHz repetition rate, and 100 ns pulse width) was utilized as the fundamental wave source. The incident laser was focused by a convex lens with a focal length of 175 mm, and the radius of the focused spot was 0.5 mm. Moreover, a broadband femtosecond laser from 900 to 1200 nm (100 kHz repetition rate, 3–7 μJ pulse energy, pulse duration <250 fs, and 150–300 mW maximum output power) was employed as a fundamental source to obtain broadband second harmonic generation from 450 to 600 nm.

## Supplementary information

Supplementary material
