# Supplementary material for: Three-dimensional nonlinear photonic crystal in naturally grown potassium–tantalate–niobate perovskite ferroelectrics
Source: Light Sci Appl. 2020 Nov 24;9:193. doi: 10.1038/s41377-020-00427-z (PMC7687908; doi:10.1038/s41377-020-00427-z)
Supplement: Supplementary file 1 — Supplementary material [file 41377_2020_427_MOESM1_ESM.docx]

**Supplementary Materials for**

**Three-dimensional nonlinear photonic crystal in naturally grown potassium-tantalate-niobate perovskite ferroelectrics**

Chang Li,^#^ Xuping Wang,^#^ Yang Wu, Fei Liang,* Feifei Wang, Xiangyong Zhao, Haohai Yu,* Huaijin Zhang

Corresponding authors:

Email: liangfei@sdu.edu.cn (Fei Liang)

Email: haohaiyu@sdu.edu.cn (Haohai Yu)

1. **Experimental section**
   1. **Growth of KTa_1-x_Nb_x_O_3_ (KTN) crystal**

Potassium tantalate niobate utilized in this experiment is grown by the Czochralski method. Chemical reagents, K_2_CO_3_, Ta_2_O_5_ and Nb_2_O_5_ with 99.99% purity, were used as raw materials in the crystal growth. Based on the phase diagram, the raw materials, which were weighed out in proportion to the ratio of desired composition of the grown crystal. They were well mixed and pressed into pieces at about 10^8^ Pa. The pieces were placed in a Pt crucible and heated at about 1100 degrees Celsius for 15 h - 20 h. The raw materials reacted, and polycrystalline KTN was obtained in accordance with the following reaction.

$$K_{2}CO_{3}+x{Nb}_{2}O_{5}+\left( 1-x \right){Ta}_{2}O_{5}=2K{Ta}_{(1-x)}{Nb}_{x}O_{3}+CO_{2}\uparrow$$

In order to grow tetragonal KTN crystal with near room Curie temperature, Ta/Nb ratio, which is ${Ta}_{2}O_{5}:{Nb}_{2}O_{5}\left( mol \right)=0.55:0.45$, was used to grow the crystal.

KTN single crystals were grown in a TDL-H50AC crystal-pulling apparatus. The platinum crucible was heated using a 2 kHz intermediate frequency heater. The temperature control apparatus was a EUROTHERM 818 controller/programmer with a precision of ±0.3 degrees Celsius. The crawling distance of the pulling apparatus was less than 1 μm. A rectangular cubic KTN single crystal bar with dimensions of 3×3×30 mm^3^ was used as the seed crystal. The experiments were performed in air atmosphere. The pulling rate was kept at 0.3–0.5 mm·h^-1^ after the crystal diameter reached a certain value. The crystal was rotated at a rate of 5–10 rpm during growth. After the growth was completed, the crystal was cooled to room temperature at a speed of 30-50 degrees Celsius per hour.

- 1. **X-ray photoelectron spectroscopy (XPS)**

XPS measurements were performed using a monochrome aluminum target Kα source (hν = 1486.6 eV, Thermo Fisher Scientific Escalab-250). The optimum energy resolution was 0.45 eV and the pressure in the chamber vacuum was approximately 1.7×10^-10^ mbar.

- 1. **P-E ferroelectric loop**

The room temperature ferroelectric hysteresis loops of the KTN crystals were measured by using a ferroelectric test system (RT-Precision LC, Radiant Technology). The sample size of measured KTN plate is 3×3×0.3 mm^3^. Piezo-response force microscopy was carried out using Asylum Research MFP-3D atomic force microscope at room temperature. A conductive cantilever with a spring constant of 2 N·m^-1^ and tip radius of 20 nm was used.

- 1. **D****ifferential scanning calorimetry (DSC)**

By applying the Labsys^TM^ EVO TG-DTA/DSC thermal analyzer equipped with the nitrogen flow at a rate of about 20 mL·min^-1^. Appropriate amounts of the polycrystalline powder were thoroughly ground, then were placed in a silica tube (5 mm o.d. × 3 mm i.d.) and subsequently sealed under a high vacuum. The tube with KTN was heated from 10 to 200 degrees Celsius and then cooled to room temperature with the heating/cooling rate both at 5 degrees Celsius per minute.

- 1. **Second harmonic generation experiment**

A piece of x-cut KTN crystal with dimensions of 0.4 mm × 4 mm × 4.5 mm (x×y×z) was employed and the two y-z faces were polished. A Nd:YAG laser (1064 nm, 20 kHz repetition rate; 100 ns pulse width) was utilized as the fundamental wave source. The incident laser was focused by a convex lens with a focal length of 175 mm and radius of the focused spot was about 0.5 mm which is far smaller than sample but covers plenty of supercells. There was a significant SHG light scattering when the fundamental wave passed through the KTN sample. Meanwhile, a broadband femtosecond laser from 900 nm to 1200 nm (repetition rate 100 kHz, pulse energy 3~7 μJ, pulse duration < 250 fs; Max output power 150~300 mW) was utilized as fundamental sources to obtain broadband SHG from 450 nm (blue light) to 600 nm (red light).

1. **Nonlinear optical coefficient tensor transformation**

Because of the modulation of ferroelectric domain to the nonlinear coefficient and the three-dimensional distribution of ferroelectric domain, nonlinear susceptibility coefficient is also distributed periodically in the three-dimensional space in the KTN crystal. In a square supercell, ferroelectric domains can be divided into four regions whose polarization directions are perpendicular to each other, namely 90°, 180°, 270°, 360°. The nonlinear susceptibility coefficients of every region are as follow:

$d_{ij}^{90^{\circ}}=\left( \begin{matrix} 0 & 0 & 0 \\ -d_{31} & -d_{31} & -d_{33} \\ 0 & 0 & 0 \end{matrix}\begin{matrix} 0 & d_{15} & 0 \\ 0 & 0 & 0 \\ d_{15} & 0 & 0 \end{matrix} \right)$ (S1)

$d_{ij}^{180^{\circ}}=\left( \begin{matrix} 0 & 0 & 0 \\ 0 & 0 & 0 \\ -d_{31} & -d_{31} & -d_{33} \end{matrix}\begin{matrix} 0 & d_{15} & 0 \\ {-d}_{15} & 0 & 0 \\ 0 & 0 & 0 \end{matrix} \right)$ (S2)

$d_{ij}^{270^{\circ}}=\left( \begin{matrix} 0 & 0 & 0 \\ d_{31} & d_{31} & d_{33} \\ 0 & 0 & 0 \end{matrix}\begin{matrix} 0 & d_{15} & 0 \\ 0 & 0 & 0 \\ {-d}_{15} & 0 & 0 \end{matrix} \right)$ (S3)

$d_{ij}^{360^{\circ}}=\left( \begin{matrix} 0 & 0 & 0 \\ 0 & 0 & 0 \\ d_{31} & d_{31} & d_{33} \end{matrix}\begin{matrix} 0 & d_{15} & 0 \\ d_{15} & 0 & 0 \\ 0 & 0 & 0 \end{matrix} \right)$ (S4)

According to $P_{i}^{2\omega}=\sum\chi_{ijk}^{(2)}\cdot E_{j}^{\omega}\cdot E_{k}^{\omega}$, the total SHG polarization *P*_2_ and *P*_3_ can be written as:

$P_{2}^{2w}=\left| P_{2,90^{\circ}} \right|+\left| P_{2,180^{\circ}} \right|+\left| P_{2,270^{\circ}} \right|+\left| P_{2,360^{\circ}} \right|=4d_{15}E_{2}E_{3}+2d_{31}{E_{2}}^{2}+2d_{33}{E_{3}}^{2}$ (S5)

$P_{3}^{2\omega}=\left| P_{3,90^{\circ}} \right|+\left| P_{3,180^{\circ}} \right|+\left| P_{3,270^{\circ}} \right|+\left| P_{3,360^{\circ}} \right|=4d_{15}E_{2}E_{3}+2d_{31}{E_{2}}^{2}+2d_{33}{E_{3}}^{2}$ (S6)

Clearly, the polarization intensity $\vec{P}_{2}$ was equal to $\vec{P}_{3}$. The SHG beam has two polarization states at the same time and the total SHG polarization intensity is the superposition of two polarization intensities,

$P_{SHG}\propto\left( \left| \cos\varphi\right|+\left| \sin\varphi\right| \right)^{2}$ (S7)

where $\varphi$ is the angle between polarizer *P*2 and z-axis.

1. **The SHG spot simulation**

Different types of quasi-phase-matching existed in KTN super-crystal are simulated in **Figure 3**. The detailed analysis is as follow:

**Fig. 3b** is a nonlinear Raman-Nash diffraction common in nonlinear photonic crystals. According to the momentum conservation condition of the nonlinear optical process:$\Delta\vec{k}=\vec{k}_{2w}{-2\vec{k}}_{w}-\vec{G}_{i,j}=0$, in which $\vec{G}_{i,j}$is the linear superposition of $\vec{G}_{\pm100}$ and $\vec{G}_{0\pm10}$ to compensate the phase mismatch along different directions, we can calculate the scattering angle θ of SHG spot as following relation:

$\cos\theta=\frac{2k_{\omega}}{k_{2\omega}}=\frac{n_{\omega}}{n_{2\omega}}$ (S8)

where $k_{\omega}$ and $k_{2\omega}$ are fundamental and frequency-doubling light wave vector, and $n_{\omega}$ and $n_{2\omega}$ are the refractive index of fundamental wave and second-harmonic wave, respectively.

**Fig. 3c** illustrates the SHG spot in a collinear quasi-phase matching phenomenon when RLV provided by supercell is collinear with the fundamental wave vector. In the process, SHG wave vector is parallel to fundamental wave and the scattering angle $\theta$ of SHG spot is zero.

**Fig. 3d** and **Fig. 3e** show the SHG spot generated by the interaction between the diffracted fundamental wave and the non-collinear super lattice RLVs in different directions, which generates diffracted light with different scattering direction. In both cases, the quasi-phase-matching condition is satisfied:

$\vec{k}_{2w}{-2\vec{k}}_{wD}-\vec{G}_{i,j}=0$ (S9)

The scattering angle $\theta$ of SHG spot in these cases can be calculated by relation:

$\cos\theta=\frac{2k_{\omega}}{k_{2\omega}}=\frac{n_{\omega}}{n_{2\omega}}$ (S10)

**Fig. 3f, Fig. 3g** and **Fig. 3h** show the SHG spot generated by the interaction between the diffracted fundamental wave and the collinear super lattice RLVs. Due to the different superposition modes of diffracted fundamental wave, these processes produce three SHG waves with different scattering angle $\theta$ while satisfying the momentum conservation condition of quasi-phase matching:

$\Delta\vec{k}=\vec{k}_{2w}{-2\vec{k}}_{wD}-\vec{G}_{k}=0$ (S11)

According to the QPM diagram shown in **Fig. 3f, Fig. 3g** and **Fig. 3h**, the angle $\theta$ under different conditions can be obtained:

$\sin\theta=\frac{k_{\omega}\sin\theta_{D}}{k_{2\omega}}=\frac{n_{\omega}}{{2n}_{2\omega}}\sin\theta_{D}$ (S12)

$\sin\theta=\frac{2k_{\omega}\sin\theta_{D}}{k_{2\omega}}=\frac{n_{\omega}}{n_{2\omega}}\sin\theta_{D}$ (S13)

where $\theta_{D}$ is the divergent angle of the diffracted fundamental wave caused by supercell grating. Equation (S12) and (S13) represent the scattering angle $\theta$ of SHG produced in quasi-phase-matching process of **Fig. 3f** and **Fig. 3h**, respectively. **Fig. 3g** is the case in which the superposition vector of diffracted fundamental wave is in same direction of incident fundamental wave vector, resulting in the same effect as collinear quasi-phase-matching.

The refractive index of fundamental wave ($n_{\omega}$) and second-harmonic wave ($n_{2\omega}$) employed in simulation were calculated by the following dispersion equation:$n^{2}-1=A+\frac{B\lambda^{2}}{\lambda^{2}-C}$, A=1.389, B=2.404, C=6.003.

Supposing the fundamental laser beams with the wavelength of 1064 nm is Gaussian distribution, the theoretical SHG pattern can be calculated using the following equation:

$I_{2w}=C^{2}\left( \sum_{\theta} e^{-\frac{\left( x+z\times tan\theta\right)^{2}+y^{2}}{r^{2}}}+e^{-\frac{x^{2}+\left( y+z\times tan\theta\right)^{2}}{r^{2}}} \right)\times Sinc\left( \frac{1}{2}\left| G \right|L \right)$ (S14)

where $I_{2w}$ is the intensity of the SHG beam, *C* is the coefficient related to the crystal parameters, *x* and *y* are coordination in the cross section of the Gaussian beams, θ is the scattering angle of SHG spot, *r* is the radius of the generated Gaussian beam, and *L* is the propagation length. θ can be calculated as listed in **Table S1**.

**Table. S1** Calculated and measured angle of the second harmonic spots via the QPM experiencing linear and nonlinear interaction.

| Pattern in **Figure 3** | Experiment | Simulation |
| --- | --- | --- |
| **b** | 12.70° | 11.09° |
| **c** | 0° | 0° |
| **d** | 37.00° | 37.67° |
| **e** | 37.00° | 37.67° |
| **f** | 21.70° | 16.86° |
| **g** | 0° | 0° |
| **h** | 31.00° | 35.45° |

1. **Additional Figures and Tables**

**4.1 X-ray photoelectron spectroscopy**


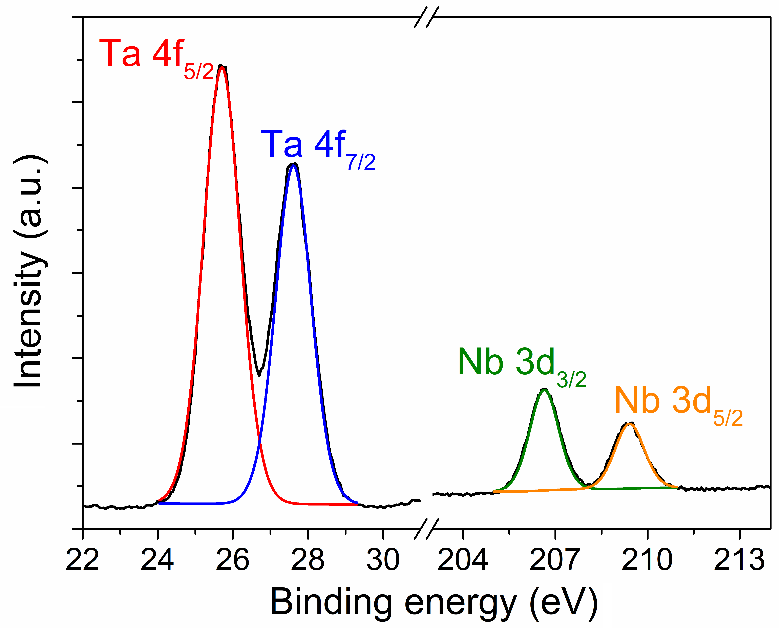


**Figure S1.** X-ray photoelectron spectroscopy (XPS) of as-grown KTN crystal

**4.2 The hysteresis loop measurement**


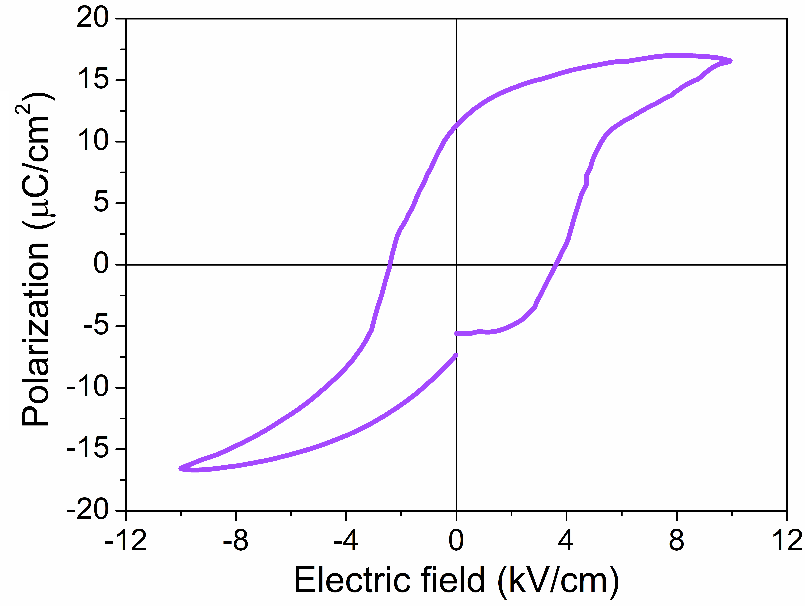


**Figure S2.** P-E hysteresis loop of as-grown KTN crystal

**4.3** **Differential scanning calorimetry measurement**


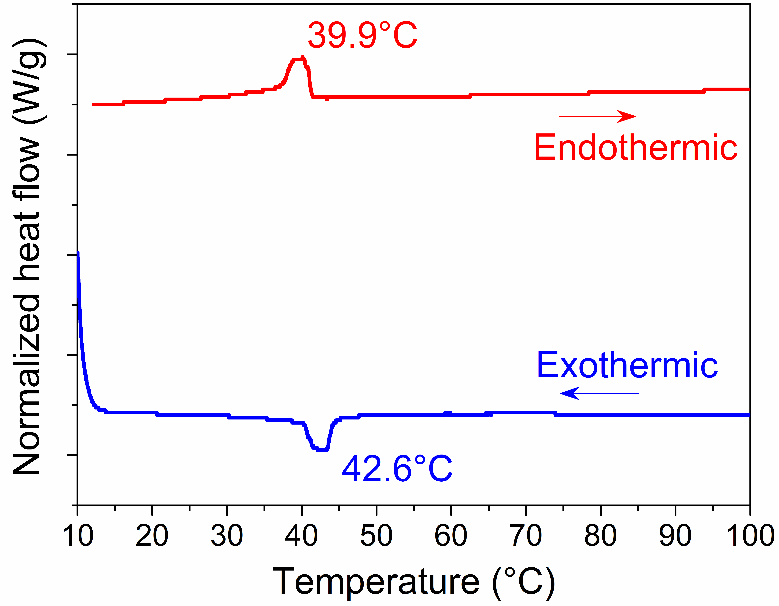


**Figure S3.** Differential scanning calorimetry (DSC) curve of as-grown KTN crystal

**4.4 Piezo-response force microscopy (PFM) measurement**


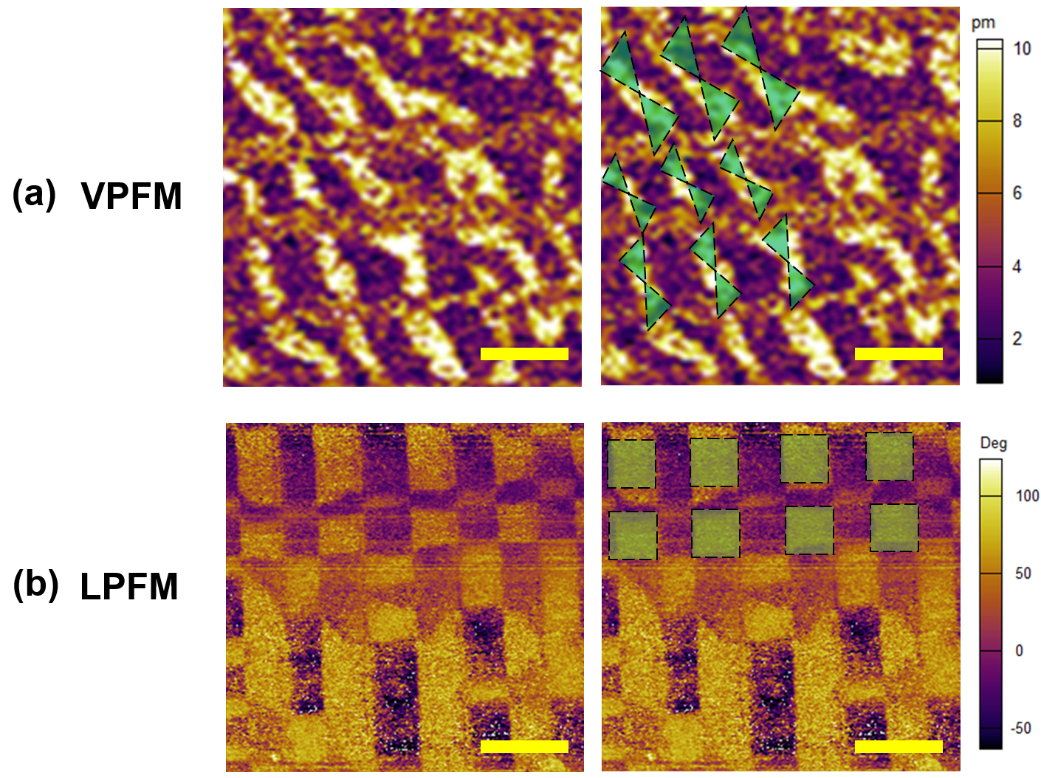


**Figure S4.** **a**, The vertical PFM amplitude and **b**, lateral PFM phase images of KTN ab-plane in the same region. The scale bar is 2 μm.

**4.5 SH image measurement**


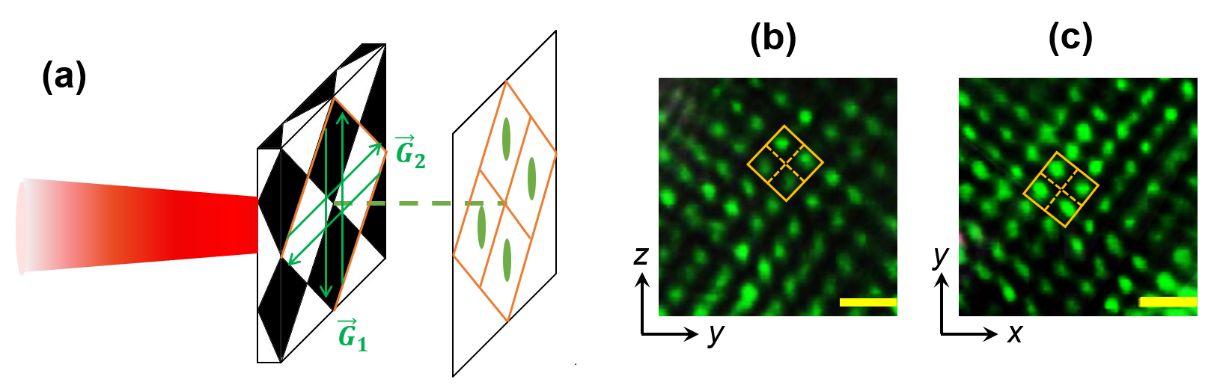


**Figure S5.** **a**, The diagram of SH spot (black and white region represent the area of inverted periodic 180° ferroelectric domains with orthogonal polarization orientation). **b-c**, The SH pattern of x-cut KTN sample and z-cut KTN sample (the scale bar is 25 μm)

As shown in **Figure S5a**, the inverted periodic 180° ferroelectric domains in black and white regions can provide reciprocal lattice vectors along two orthogonal directions, which leads to effective SH light (green spot in **Figure S5b** and **S5c**) in both horizontal and vertical direction. In contrast, the boundary of the orthogonal ferroelectric domain region along the 45° direction is 90° domain walls, which provides no significant reciprocal lattices to obtain high SH intensity, thereby resulting in dark region in **Figure S5b** and **S5c**. In another word, the total SH spot is divided into neat squares (green spot, 180° domains) by 90° domain walls (dark region). Therefore, we can draw a statement that the green spots in the SH image represent the 180° domains and the dark region represents 90° domain walls.

**4.6 Statistical calculations of supercells on SH image**


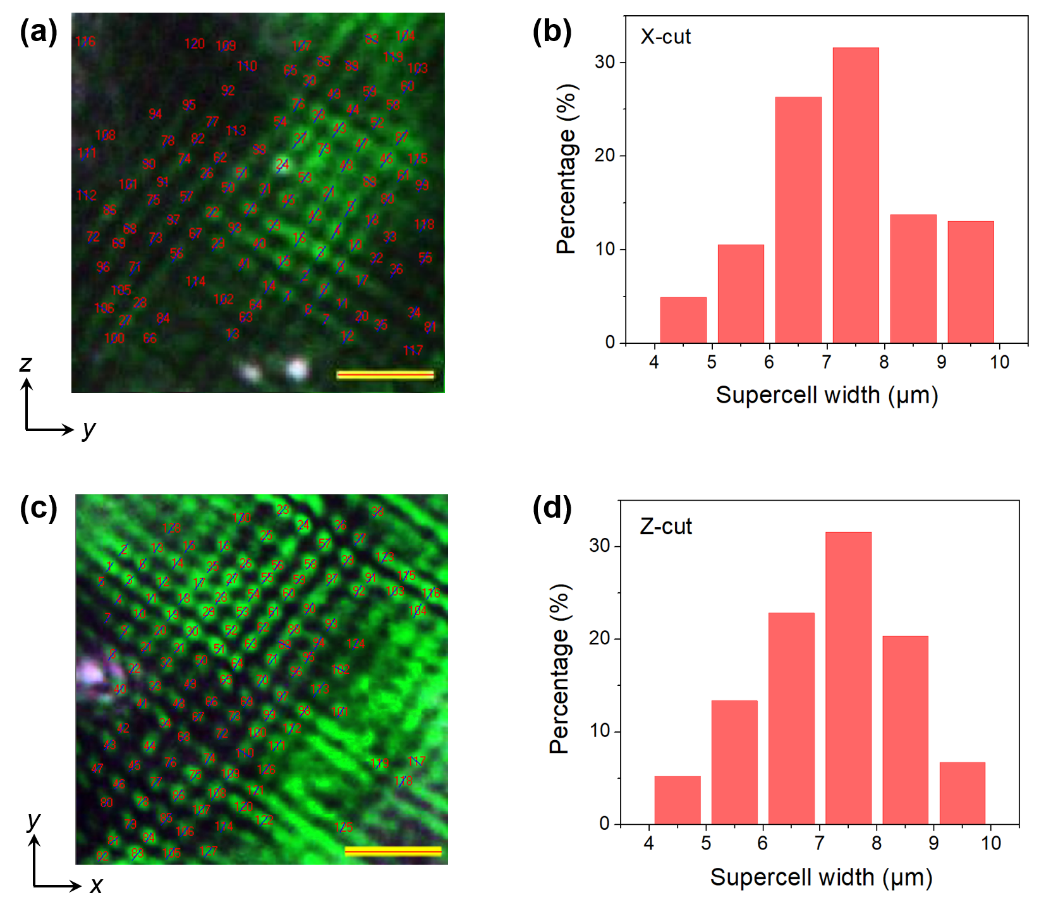


**Figure S6.** **a, b**, statistical distributions of supercell in x-cut KTN crystal; **c, d**, statistical distributions of supercell in x-cut KTN crystal.

The green spots in SH image represent a couple of inverted 180° ferroelectric domains. Therefore, the width of the SH spots, equaling to twofold inverted periodic 180° ferroelectric domain width, could be set as the supercell constant. The “*Nano-measurer*” software was employed to measure the size of SH spots in the near-field SH image. First, we set a scale bar of 50 μm (yellow line on the bottom right corner) to define the overall size of SH graph. Then, we draw blue lines to pick the longest width of every SH spots, as the respective size of coupled 180° ferroelectric domains, also the constant of supercells. We plotted 120 and 127 blue lines in **Figure S6a** and **S6c**, respectively. On the basis of the software measurement, the statistical results of supercell width of x-cut and z-cut crystal were counted in **Figure S6b** and **S6d**. The narrowest and widest supercells have the widths of 3.4 μm and 9.6 μm.

**4.7 Broadband SHG spectrum**


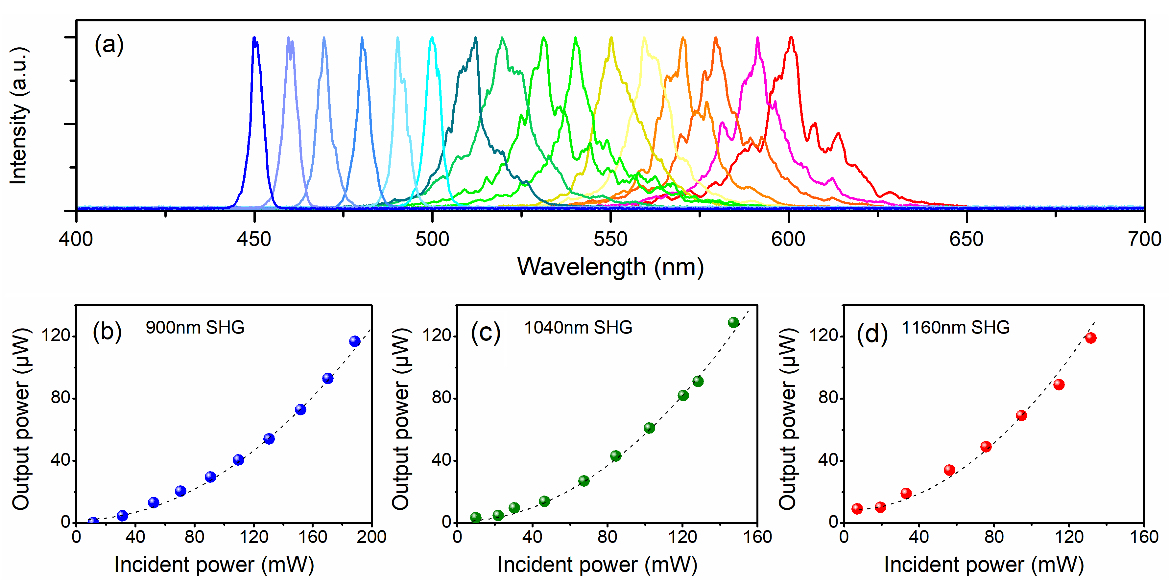


**Figure S7.** **a**, The broadband SHG spectrum of x-cut KTN crystal; **b**, **c**, **d**, the SHG power versus incident power at 900 nm, 1040 nm and 1160 nm.
